# Low‐ Temperature Transformations in Amorphous Silica Bilayers on Ru(0001) After Crystal‐Glass Transition: Closer Look

**DOI:** 10.1002/chem.202502669

**Published:** 2025-10-07

**Authors:** Leonard Gura, Ya‐Fan Chen, Marek Sierka, Markus Heyde, Zechao Yang, Hans‐Joachim Freund

**Affiliations:** ^1^ Fritz‐Haber‐Institut der Max‐Planck‐Gesellschaft Faradayweg 4–6 14195 Berlin Germany; ^2^ Otto Schott Institute of Materials Research Friedrich Schiller University Jena Löbdergraben 32 07743 Jena Germany

**Keywords:** density functional calculations, phase transitions, scanning tunneling microscopy, silica bilayers, vitreous bilayers

## Abstract

The crystal‐glass transition concerning silicon dioxide has been a topic of intense research. It has been possible to prepare a bilayer silica film on a metal substrate and study the corresponding transition in real space using scanning tunneling microscopy (STM). While the initial trigger of the transition has been identified as a so‐called Stone–Wales ring‐opening process, which requires rather high energies/temperatures, a question has been, whether there are similar follow‐up processes, which require much less energy and thus rather moderate temperatures. Although observations have been reported using electron microscopy, it remains unclear whether, in this case, the modifications were initiated by the measurement itself. Here, we report on a fast‐scanning STM study, allowing a study in real space and indicating structural transformations at room temperature. The experimental results are supported by demanding density functional calculations, which allow for an understanding of the involved ring structure modifying processes. The calculations also reveal the influence of the metal support on the transformation energetics.

## Graphical abstract

In this work we report on the ring opening of the silica film as studied by fast‐scanning scanning tunneling microscopy together with density functional calculations. The results reveal the influence of the metal support on the transformation energetics.

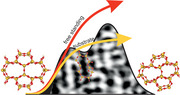

## 1 Introduction

**Figure 1:**
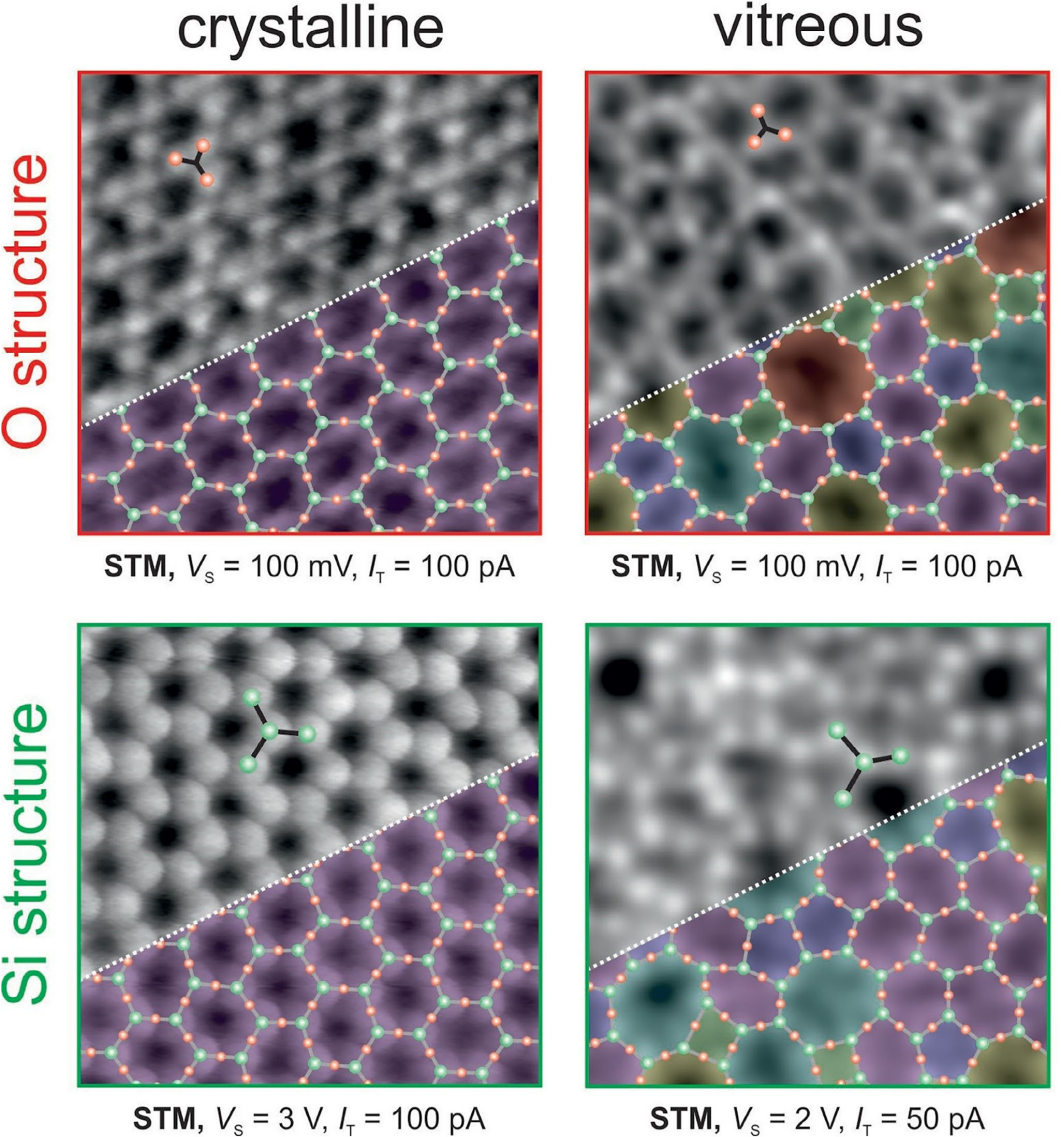
STM images of a crystalline and vitreous bilayer silica film taken with different tunnelling conditions, which emphasize in one case the O‐atom based substructure, and in the other case the Si‐atom based substructure. The scan area of all images is 3.5 nm × 3.5 nm.^[^ ^2^ ^]^

The 2D silica bilayer on a metal support^[^ ^1^ ^]^ has been widely studied as a model system for understanding the crystal‐glass transition process. The direct transformation from crystalline to vitreous phases (see Figure 1) is characterized by a high activation energy and involves intermediate structures with lower activation energies.

Previous studies using Low Energy Electron Microcopy (LEEM), as a function of temperature, have reported a Stone–Wales type defect transformation from four six‐membered rings, by the way, the only ring size in the crystalline material, into a set of two five‐ and two seven‐membered rings in the vitreous structure, as the rate determining step for the crystal‐glass transition, with an activation energy of around 4 eV, consistent with the high transition temperature of near 1000 K.^[^ ^3^ ^]^ The structural transformations and the activation energies associated with the formation of other ring sizes, and structural changes remain unclear. However, this is a particularly interesting issue, as it is connected with the question whether the glass structure is frozen at lower temperatures. There are strong indications from in‐situ neutron scattering on 3D samples for continuous structural changes below the crystal‐glass transition temperature,^[^ ^4^ ^]^ but there are no direct observations in real space. Structural modifications with atomic resolution in real space have been reported under electron radiation in an electron microscope for the 2D silica films.^[^ ^5^ ^]^ However, there has been no reports, so far, where direct observations of structural modifications in vitreous silica have been observed at close to room temperature without external impact concerning energies typically used in electron microscopy. Within the present study, we report first results of such observations using scanning tunneling microscopy in a high‐speed scanning mode and we investigated the structure and defect formation mechanism of amorphous SiO_2_ bilayers using 2D periodic density functional theory (DFT) calculations. The results show that the charge transfer between the amorphous SiO_2_ bilayer and the Ru(0001) surface effectively lowers the activation energy required for defect formation when compared to a free‐standing amorphous SiO_2_ bilayer.

## 2 Experimental Methods

A silica bilayer is deposited on a Ru(0001) single crystal. The Ru(0001) single crystal was cleaned by repeating cycles of Ar^+^‐ion bombardment, annealing in ultra‐high vacuum (UHV) and in oxygen atmosphere (3 × 10^−6^ mbar) at 1300 to 1400 K. The silica layer is produced by evaporation of silicon in an oxygen atmosphere of 2.3 × 10^−7^ mbar and subsequent annealing in oxygen (2.2 × 10^−6^ mbar) at 1225 K. The sample is cooled in the same oxygen atmosphere.

High‐speed STM measurements are performed at room temperature in UHV at a base pressure in the range of 10^−10^ mbar. The STM uses spiral patterns for high‐speed imaging.^[^ ^6^, ^7^ ^]^

## 3 Theoretical Methods

All DFT calculations were performed using the TURBOMOLE^[^ ^8^, ^9^ ^]^ program package. Geometry optimizations for all structures employed 2D periodic boundary conditions in combination with the PBE exchange‐correlation functional^[^ ^10^ ^]^ and the pob‐TZVP‐rev2 basis set.^[^ ^11^ ^]^ For Ru atoms, an effective core potential (ECP) was applied, and long‐range dispersion interactions were treated using Grimme's DFT‐D3 correction with Becke–Johnson damping.^[^ ^12^, ^13^ ^]^ The resolution of identity approximation and the continuous fast multipole method were applied to the Coulomb term,^[^ ^14^, ^15^ ^]^ together with the corresponding auxiliary basis sets.^[^ ^16^ ^]^ Reaction pathways were optimized at the same theoretical level using chain‐of‐states methods as implemented in the Woelfling module of TURBOMOLE.^[^ ^17^ ^]^

Convergence of pathway optimizations was monitored through the mean root‐mean‐square value of the gradient along the path. For the free‐standing amorphous SiO_2_ bilayer, the default convergence threshold of 1.0 × 10^−4^ was used. For the SiO_2_ bilayer supported on Ru(0001), a reduced threshold of 2.0 × 10^−4^ was applied, as the larger model size leads to considerably higher computational cost.

The slab model of Ru(0001) was constructed with a 4 × 6 supercell, with lattice parameters of *a* = 18.220 Å and *b *= 15.804 Å. The model contains five Ru layers, with the bottom three layers fixed to their bulk positions and the top two layers allowed to relax.

## 4 Results and Discussion

**Figure 2:**
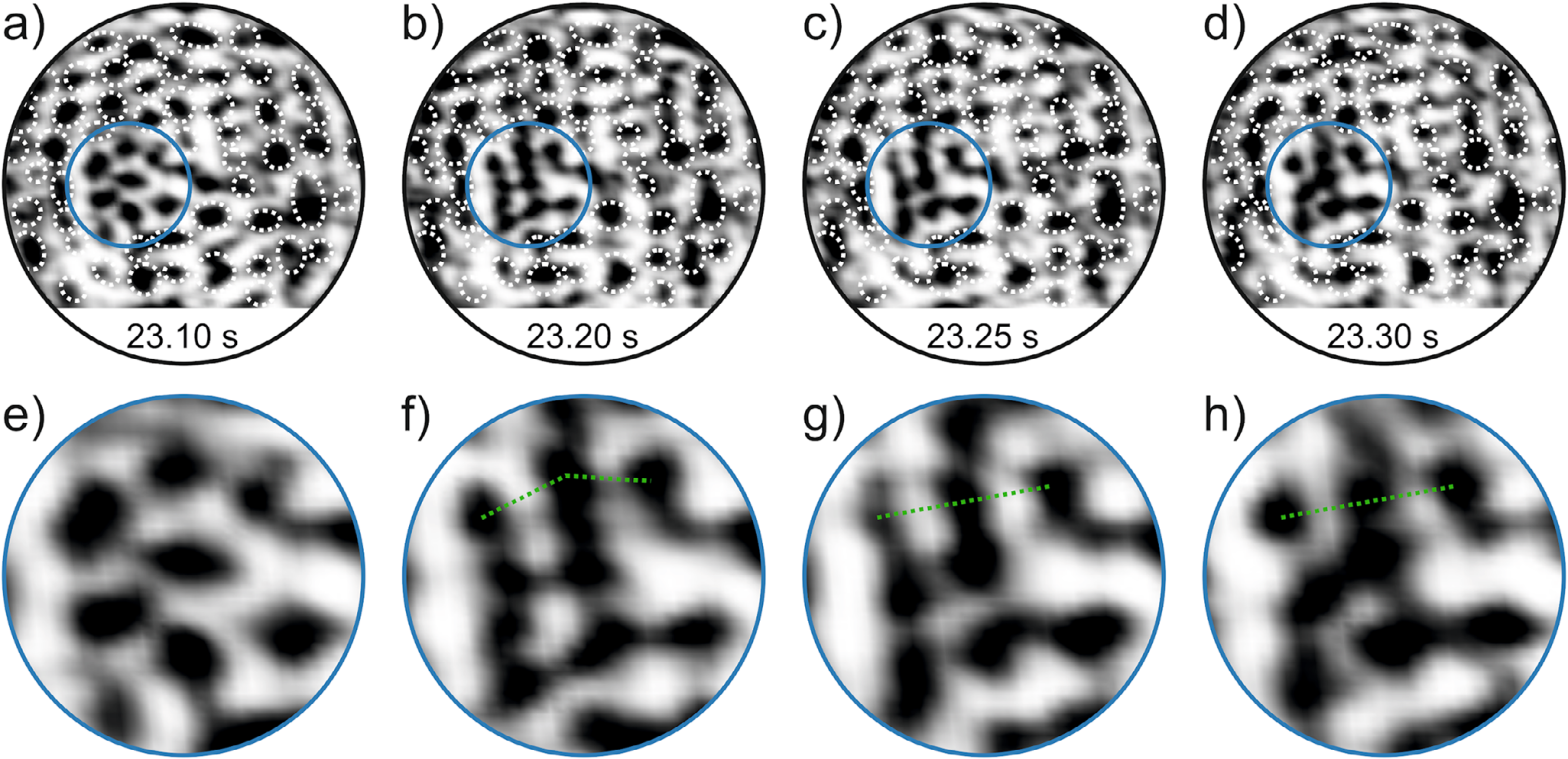
Dynamic changes of the apparent ring configuration. a–d) High‐speed spiral STM images on 2D silica (*V*_S_ = 0.8 V, *I*_T_ = 1 nA, *T* = 300 K, scan diameter = 5 nm). Each frame is acquired within 50 ms. The absolute time is indicated at the bottom of the frames and is related to the supplementary video. White dotted ellipses mark detected rings. The regions highlighted in blue are magnified in e–h). The green, dotted lines serve as guide to the eye to illustrate consistent changes in the ring alignment.

High‐speed STM images are acquired at a time resolution of 50 ms per frame, corresponding to a frame rate of 20 frames per second. The video in the Supplemental Material (Supplemental video) shows a scan sequence of approximately 27 seconds. From this scan sequence, an extraction of consecutive images is shown in Figure 2a–d. Figure 2a shows the ring configuration that appears stable for more than 20 seconds of the video. Then, rapid changes can be observed in the area highlighted in blue in Figure 2b–d. For illustration, the structural features of the unchanged areas are marked with white dotted ellipses. Figure 2e–h shows the magnified blue region at the given times. The green line illustrates that the apparent ring configuration changes over time from e to h.

In addition to the real time video, the Supplemental video shows on the right the video after post processing with the motion magnification algorithm from Wadhwa et al.^[^ ^18^ ^]^ The motion magnified video shows only mild oscillations in the first 20 seconds of the video. Then, stronger motions are detected. This coincides with the time when the apparent ring network structure changes in Figure 2. From second 25, the motion magnified video gets more and more calm. At this point, no further changes in the apparent ring structure are detected and the configuration remains as shown in Figure 2c,d.

**Figure 3:**
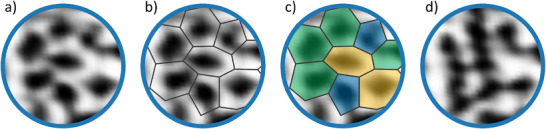
Detected ring network. a–c) show the STM image of Figure 2e) with the detected network structure. blue: five‐membered ring, green: six‐membered ring, yellow: seven‐membered ring. d) shows the STM image of Figure 2f).

To focus on the apparent ring configuration, Figure 3 shows the same region as in Figure 2e with the detected ring network structure. Figure 3b shows the outlines of the detected rings and Figure 3c shows the color‐coded ring sizes.

Figure 3d shows the apparent ring network structure also shown in Figure 2f. In this image, the ring network appears very different and shows rather elongated ring features. The image contrast cannot be mimicked with closed ring network as described in previous publications.^[^ ^19^ ^]^

If the image contrast changes from a closed ring network structure to a structure that shows rather elongated rings, the question arises whether at room temperature, dynamic changes in the silica film can be expected theoretically.

To rationalize the observed dynamics and assess whether such changes are feasible at room temperature, we computed reaction pathways on free‑standing and Ru(0001)‑supported SiO_2_ bilayers. We considered several representative ring systems and selected three low‑energy defect families, 5‑6‑5‑7, 5‑7‑6‑7, and 5‑8‑5‑8, for detailed pathway optimizations. These derive from sequences of Stone–Wales‑type transformations originating from the pristine hexagonal bilayer; the canonical 6‑6‑6‑6 to 5‑7‑5‑7 step has been established previously and is not repeated here.^[^ ^3^ ^]^

**Figure 4:**
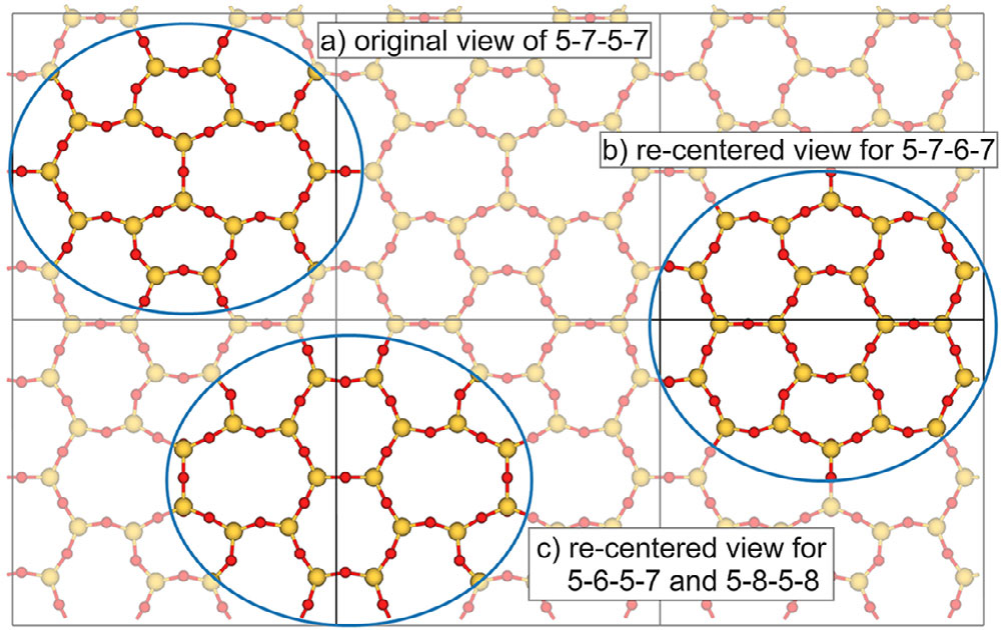
Original versus re‑centered views of the 5‑7‑5‑7 ring configuration. In the original view a), defect sites for subsequent transformations (5–6–5–7, 5–7–6–7, 5–8–5–8) lie off‑center. For clearer visualization, the structures are recentered within the surface unit cell, so that the defect sites appear near the cell center: b) recentered view for the 5–7–5–7 to 5–7–6–7 pathway; c) recentered view for the 5–7–5–7 to 5–6–5–7/5–8–5–8 pathways.

**Figure 5:**
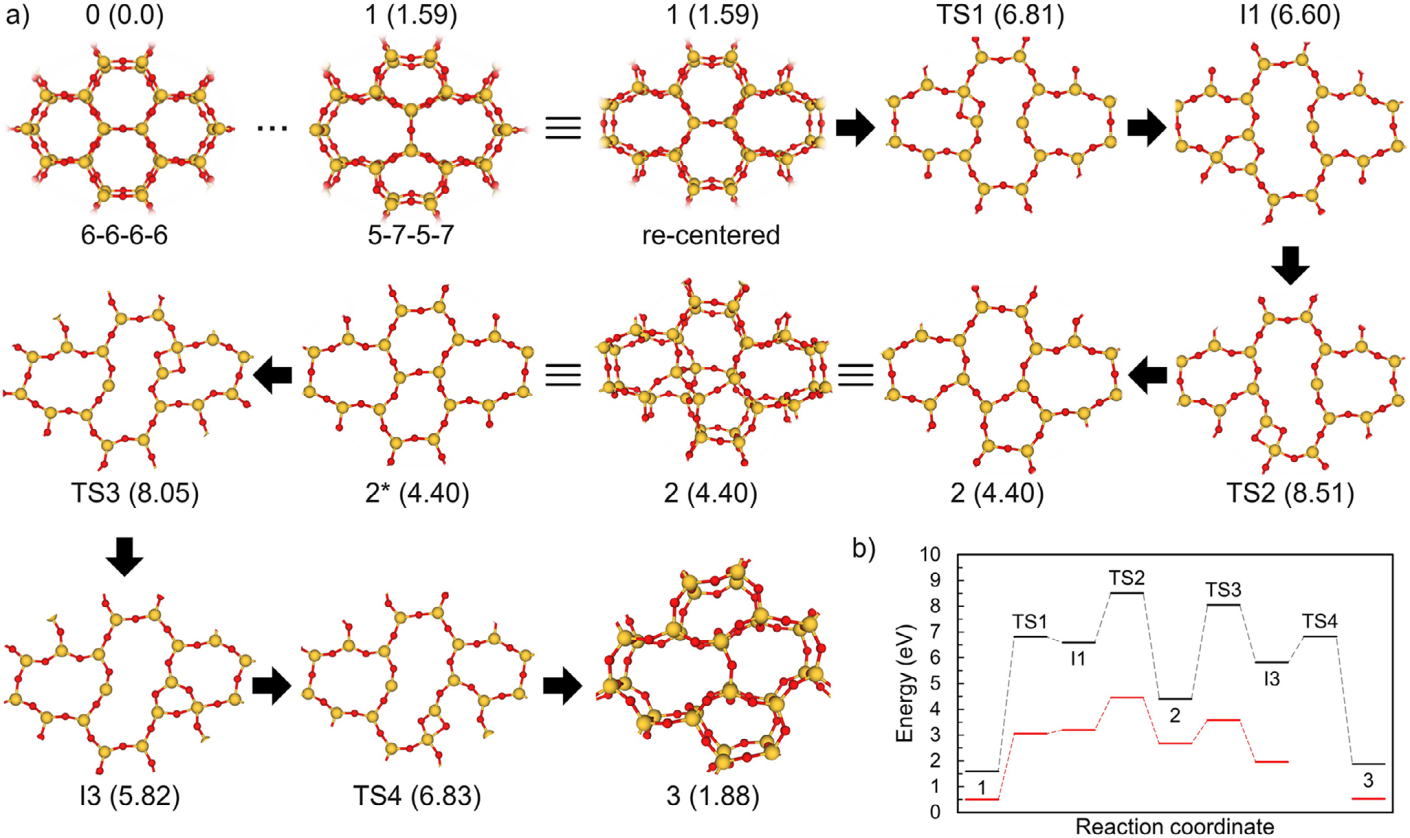
a) Transformation sequence from a 5–7–5–7 to a 5–6–5–7 ring system in a free‐standing SiO_2_ bilayer (partial view). The intermediate steps leading to structure 1 (5–7–5–7) are omitted here and can be found in detail in ref. [3]. Structure 1 is shown first in its original view and then in a recentered view to highlight the defect site, and this perspective is retained for all subsequent structures until structure 3 (5–6–5–7), which is shown again in the original view. The energies (in eV) relative to structure 0 are given in parentheses. Structure 0, 1, 2, and 3 are shown with both layers of the bilayer, while the other structures are shown as single layers for clarity. The transformation starts from the upper layer of the SiO_2_ bilayer, as shown in the single‐layer structures before structure 2, while those after structure 2* represent the opposite side of the bilayer. Si and O atoms are colored yellow and red, respectively. b) Potential energy diagram along the reaction coordinates corresponding to the transformation pathway. Black for free‐standing SiO_2_ bilayer, red for SiO_2_ bilayer supported on Ru(0001). TS4 (red) is not available.

For visualization and robust pathway optimization, all structures along a given pathway were recentered within the periodic supercell so that the defect consistently appears near the cell center (see Figure 4). Note that total energies are invariant to uniform translations of the surface unit cell. Recentering thus standardizes the viewpoint across steps and avoids numerical issues when defects approach cell boundaries (relevant for the Woelfling chain‑of‑states implementation). In the 6‑6‑6‑6 to 5‑7‑5‑7 transformation, the Stone–Wales‑type defect forms at the center of the supercell,^[^ ^3^ ^]^ whereas for the subsequent pathways (5‑6‑5‑7, 5‑7‑6‑7, 5‑8‑5‑8) the defect sites in the original views lie near the cell boundaries. Figure 4a–c illustrates original and recentered views for the 5‑7‑5‑7 structure (structure 1 in Figure 5a).

Reaction pathways were determined for transformations of ring systems from 5–7–5–7 to 5–6–5–7, 5–7–6–7, and 5–8–5–8, as illustrated in Figures 5a and S1 (Supporting Information), and Figure S2 (Supporting Information), respectively. The sequence of the 6–6–6–6 to 5–7–5–7 transformation is not included here, as it has been reported in previous work.^[^ ^3^ ^]^ Instead, Figure 5 focuses on the subsequent steps starting from the 5–7–5–7 ring system (structure 1). The transition states (TS) and intermediate (I) structures for the pathways of 5–7–5–7 to 5–6–5–7 and 5–7–5–7 to 5–7–6–7 result in the formation of a two‑membered and a three‑membered ring, respectively. However, for the pathway from 5–7–5–7 to 5–8–5–8, the TS form two two‑membered rings, and no intermediate structures (besides structure 2) were observed.

For the free‐standing structures in the reaction pathway optimization, the cell parameters were initially set as the average of the cell parameters of the initial and final states. These parameters were then fully optimized, except for those of the transition states. For the SiO_2_/Ru(0001) systems, calculations were performed on all stable structures (structure 1, I1, 2, I3, and 3) involved in the transformations from the 5–7–5–7 ring configuration to both the 5–6–5–7 and 5–7–6–7 ring systems. Due to the high computational cost of transition‑state searches for the large SiO_2_/Ru(0001) models, only three transition states (TS1, TS2, and TS3) for the 5–7–5–7 to 5–6–5–7 transformation were investigated in detail.

The potential energy diagram in Figure 5b shows the relative energies along the reaction coordinates corresponding to the transformation pathway. For the free‐standing SiO_2_ bilayer, the first transition state (TS1) exhibits an activation energy of 5.22 eV. To evaluate the influence of the substrate, further calculations were performed for the SiO_2_ bilayer supported on Ru(0001), following the same procedures and initiating the transformation from the upper layer of the SiO_2_ bilayer. This approach was selected because initiating the transformation from the bottom layer, where dispersive forces are present, is significantly more energetically demanding.^[^ ^3^ ^]^ While the calculations were performed assuming a sequential transformation, it should be noted that both layers may transform in a synchronized way in real space due to their structural coupling.

In the presence of the Ru(0001) substrate, the relative energies of the defect structures, referenced to the supported 6–6–6–6 ring configuration, are significantly reduced, indicating a stabilizing effect of the substrate. The first transition state (TS1) in Figure 5b exhibits an activation energy of 2.56 eV, while the second (TS2), required to complete the transformation of the upper SiO_2_ layer, presents an activation energy of 1.26 eV. However, to fully form the 5–6–5–7 defect, the bottom layer must undergo the same procedure. This requires two additional transition states to be overcome. For the third transition state (TS3), which initiates the transformation in the lower SiO_2_ layer, an activation energy of only 0.9 eV was found.

Comparable activation energies were found for diffusion processes of chemisorbed oxygen atoms on Ru(0001). These diffusion processes could be observed at room temperature^[^ ^20^, ^21^, ^22^ ^]^ and references therein. The exemplary activation energy of 0.9 eV for a transition state of transformations of silica rings shows that structural changes in the silica film can be expected to occur—even at room temperature.

Table 1 summarizes the calculated relative energies of all considered structures and the corresponding charge transfer (Δ$q_{SiO_{2}}$) between the SiO_2_ bilayer and the Ru(0001) substrate. According to the definition provided in the Supporting Information, a positive value of Δ$q_{\mathrm{Si}O_{2}}$ indicates a net electron transfer from the SiO_2_ bilayer to the Ru substrate. All values listed in Table 1 are positive, confirming that the overall electron flow is directed from the bilayer to the substrate, leaving the SiO_2_ layer slightly positively charged.

**Table 1:** Relative energies (in eV) relative to a 6–6–6–6 ring system in different reaction pathways, and charge transfer (Δ$q_{\mathrm{Si}O_{2}}$) between the SiO_2_ bilayer and the Ru substrate. The charge transfer of the 6–6–6–6 ring system supported on Ru(0001) is 1.13 electrons per surface unit cell. (level of theory: PBE/pob‐TZVP‐rev2).^[^ ^a^ ^]^

		1	TS1	I1	TS2	2	TS3	I3	TS4	3
5–7–5–7 to 5–6–5–7										
	Free‐standing	1.59	6.81	6.60	8.51	4.40	8.05	5.82	6.83	1.88
	SiO_2_/Ru(0001)	0.50	3.06	3.20	4.46	2.68	3.58	1.96	‐	0.53
	Δ$q_{\mathrm{Si}O_{2}}$	1.00	0.64	0.60	0.52	0.72	0.58	0.66	–	0.38
5–7–5–7 to 5–7–6–7										
	Free‐standing	1.59	6.50	6.09	7.25	3.27	6.99	5.73	6.26	1.44
	SiO_2_/Ru(0001)	0.50	–	2.38	–	1.53	–	0.72	–	1.60
	Δ$q_{\mathrm{Si}O_{2}}$	1.00	–	0.63	–	0.66	–	0.77	–	0.69
5–7–5–7 to 5–8–5–8										
	Free‐standing	1.59	11.50	–	10.70	4.05	–	–	–	1.18

^[a]^ The minus sign (−) indicates that the corresponding structure was not calculated.

Several structures exhibit relatively lower values of Δ$q_{\mathrm{Si}O_{2}}$, approximately between 0.4 and 0.6 electrons per surface unit cell. These include structures TS1, I1, TS2, TS3, and 3 in the 5–7–5–7 to 5–6–5–7 pathway (see Figure S4), and I1 in the 5–7–5–7 to 5–7–6–7 pathway (see Figure S5). In these cases, a portion of the electron density is transferred back from the Ru substrate to the SiO_2_ bilayer and localizes at the defect site. This charge redistribution contributes to the stabilization of the defect configuration and plays an important role in lowering the activation barriers along the transformation sequence.

In contrast, several other structures, such as structure 1, 2, and I3 in the 5–7–5–7 to 5–6–5–7 pathway, and structure 1, 2, I3, and 3 in the 5–7–5–7 to 5–7–6–7 pathway exhibit relatively higher charge transfer values, approximately between 0.7 and 1.0 electrons per surface unit cell. In these structures, a small portion of the electron density is still transferred back from the substrate into the bilayer and is partially localized within the SiO_2_ bilayer. However, since this effect is less pronounced than in the previously discussed cases, this results in higher net charge transfer values.

This charge redistribution process is closely linked to the stabilization of each defect configuration. For example, in the 5–7–5–7 to 5–6–5–7 transformation, the intermediate structure I3 (1.96 eV) has a lower relative energy than structure 2 (2.68 eV) when supported on Ru(0001) (see Figure 5b or Table 1). This stabilization can be attributed to a combination of charge transfer and a favorable registry between the SiO_2_ bilayer and the Ru substrate.

Our results also indicate that the registry between the SiO_2_ bilayer and the Ru substrate strongly influences the final relative energies. The lowest relative energies are obtained when O atoms in the bilayer are positioned directly above Ru atoms in the top layer, and Si atoms align with Ru atoms in the second layer. However, perfect alignment of all Si atoms with Ru atoms in the second Ru layer is geometrically unfeasible. For example, in the 6–6–6–6 ring system supported on Ru(0001), only three Si atoms in the six‐membered rings can be positioned above the Ru atoms in the second Ru layer. In a previous study we showed the coexistence of different registries to the substrate.^[^ ^23^ ^]^

The three exemplary structural transformations in Figures 5, S1 and S2 show that a large variety of structural changes are possible. The activation energies vary from below 1 eV to more than 4 eV and are influenced by the substrate and the registry to the substrate. Of course, other factors such as imperfections in the substrate material or the film itself will have a big impact on activation energies of local structural changes. Every transition and intermediate states in Figures 5, S1, and S2 show some kind of elongated ring feature.

**Figure 6:**
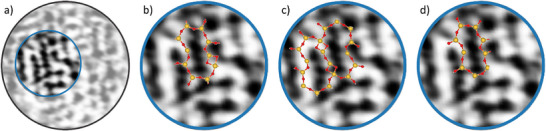
Overlaid ring configurations from theory on experimental STM data for visualization. a) STM image frame from Figure 2b. b) with fraction of the structure of TS3 from Figure 5a. c) with fraction of the structure TS2 of Figure S2a. d) with fraction of the structure of I3 from Figure S1a.

When comparing these ring configurations with the experimental STM data in Figure 3, similar elongated features are observed, as illustrated in Figures 3 and 5. To better illustrate this similarity, Figure 6 shows selected elongated ring structures from the theoretical models overlaid on the experimental STM image. The overlaid structures include fragments of TS3 from Figure 5a, TS2 from Figure S2a, and I3 from Figure S1a. These visual comparisons further support that both the experimental and theoretical results consistently show elongated ring features.

## 5 Conclusion

Dynamic changes in apparent ring configurations in a silica bilayer were detected in real space and real time. To evaluate the experimental result, we have studied the transformation process of different defects formed through a series of Stone–Wales type transformations with DFT calculations. Three‐membered rings were found in the intermediate structures from the reaction pathways of 5–7–5–7 to 5–6–5–7 and 5–7–6–7 to be an energetically favorable ring configuration. Moreover, the long‐range cooperativity between defect sites can also assist in the dissipation of strain within the SiO_2_ bilayer. Of particular significance is the charge density transfer between the SiO_2_ bilayer and the Ru substrate, which has been observed to facilitate the stabilization of the structures and effectively reduce the activation energies compared to a free‐standing SiO_2_ bilayer. The reduction of the activation energy for structural changes can explain that locally structural changes at room temperatures in the silica film are feasible.

## Conflict of Interest

The authors declare no conflict of interest.

## Supporting information



Supporting Information

Supporting Information

## Data Availability

The data that support the findings of this study are available from the corresponding author upon reasonable request.
