# Supplementary material for: Low‐ Temperature Transformations in Amorphous Silica Bilayers on Ru(0001) After Crystal‐Glass Transition: Closer Look
Source: Chemistry. 2025 Oct 7;31(61):e02669. doi: 10.1002/chem.202502669 (PMC12587026; doi:10.1002/chem.202502669)
Supplement: Supplementary file 1 — Supporting Information [file CHEM-31-e02669-s002.pdf]

Supporting Information for

"Low Temperature Transformations in Amorphous Silica Bilayers on Ru(0001) after Crystal-Glass Transition: Closer Look"

Leonard Gura,[a] Ya-Fan Chen,[b] Marek Sierka,*[b], Markus Heyde[a], Zechao Yang[a] and Hans-Joachim Freund*[a]

[a] Dr. L. Gura, Dr. M. Heyde, Dr. Z. Yang, Prof. H.-J. Freund
Fritz-Haber-Institut der Max-Planck-Gesellschaft
Faradayweg 4–6, 14195 Berlin, Germany
E-mail: freund@fhi.mpg.de

[b] Y.-F. Chen, Prof. M. Sierka,
Otto Schott Institute of Materials Research
Friedrich Schiller University Jena
Löbdergraben 32, 07743 Jena, Germany
E-mail: marek.sierka@uni-jena.de

Table of Contents

Computational Details S2
Charge Density Analysis S5
Planar Averaged Charge Density Profiles in Z Direction S12
Experimental Section S13
References S13

Computational Details

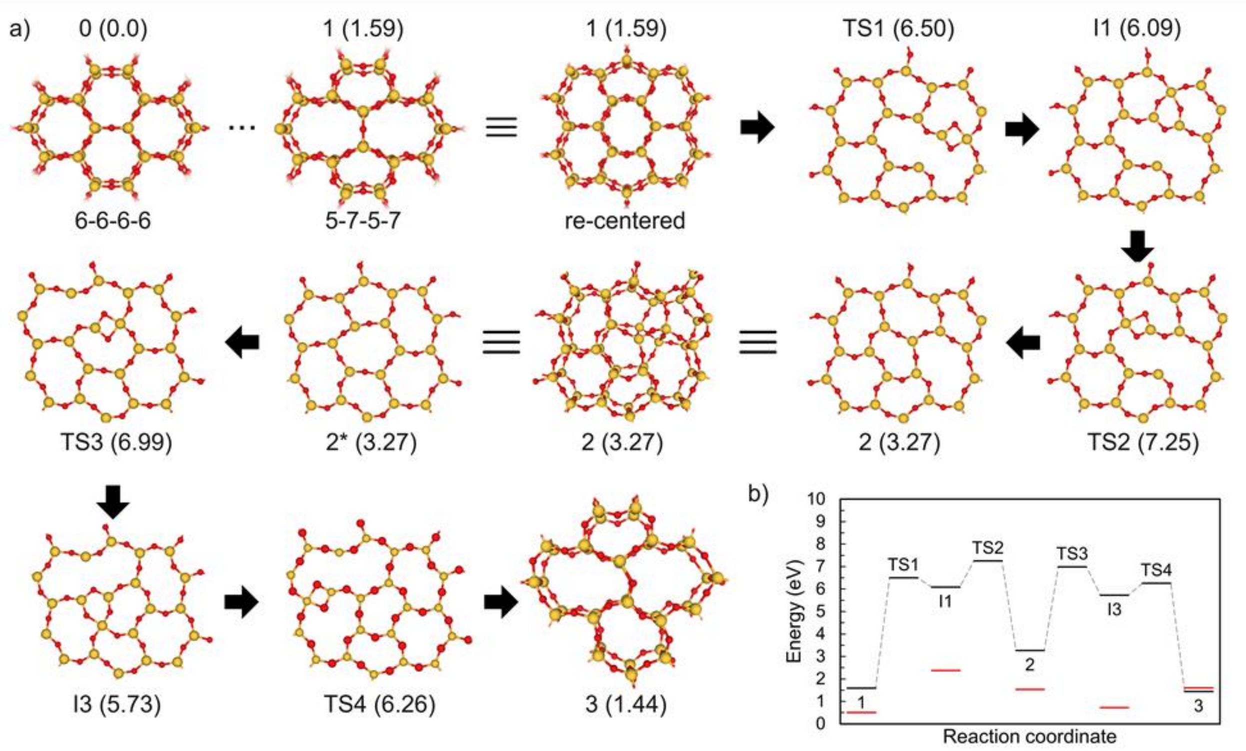

Figure S1. a) Transformation sequence from a 5-7-5-7 to a 5-7-6-7 ring system in a free-standing SiO_2 bilayer (partial view). The intermediate steps leading to structure 1 (5-7-5-7) are omitted here and can be found in detail in ref.[1]. Structure 1 is shown first in its original view and then in a re-centered view to highlight the defect site, and this perspective is retained for all subsequent structures until structure 3 (5-7-6-7), which is shown again in the original view. The energies (in eV) relative to structure 0 are given in parentheses. Structure 0, 1, 2, and 3 are shown with both layers of the bilayer, while the other structures are shown as single layers for clarity. The transformation starts from the upper layer of the SiO_2 bilayer, as shown in the single-layer structures before structure 2, while those after structure 2* represent the opposite side of the bilayer. Si and O atoms are colored yellow and red, respectively. b) Potential energy diagram along the reaction coordinates corresponding to the transformation pathway. Black for free-standing SiO_2 bilayer, red for SiO_2 bilayer supported on Ru(0001).

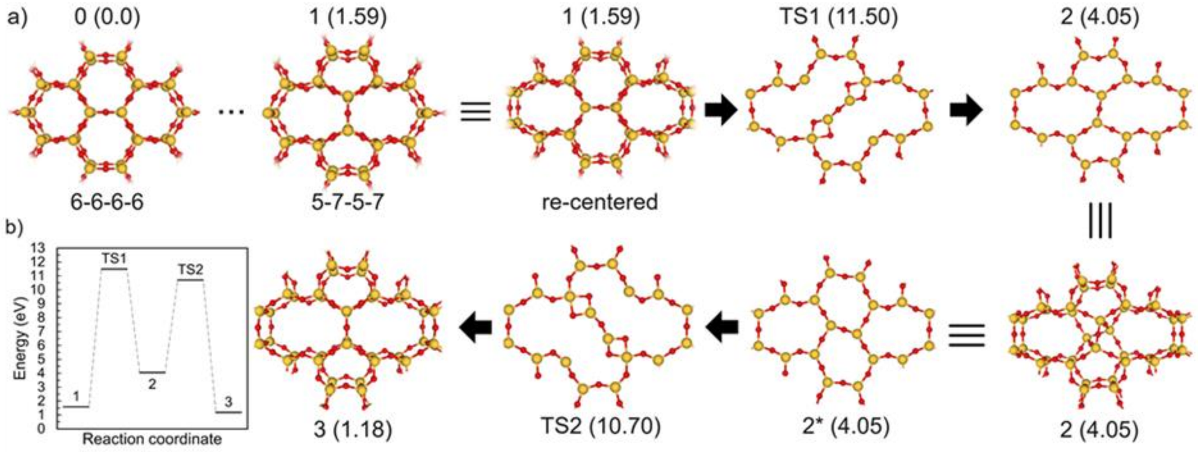

Figure S2. a) Transformation sequence from a 5-7-5-7 to a 5-8-5-8 ring system in a free-standing SiO_2 bilayer (partial view). The intermediate steps leading to structure 1 (5-7-5-7) are omitted here and can be found in detail in ref.[1]. Structure 1 is shown first in its original view and then in a re-centered view to highlight the defect site, and this perspective is retained for all subsequent structures until structure 3 (5-8-5-8), which is shown again in the original view. The energies (in eV) relative to structure 0 are given in parentheses. Structure 0, 1, 2, and 3 are shown with both layers of the bilayer, while the other structures are shown as single layers for clarity. The transformation starts from the upper layer of the SiO_2 bilayer, as shown in the single-layer structures before structure 2, while those after structure 2* represent the opposite side of the bilayer. Si and O atoms are colored yellow and red, respectively. b) Potential energy diagram along the reaction coordinates corresponding to the transformation pathway for free-standing SiO_2 bilayer.

Table S1. Optimized cell parameters for stable structures and averaged cell parameters for transition states in a free-standing SiO_2 bilayer (level of theory: PBE/pob-TZVP-rev2)

	Relative energy (eV)	*a* (Å)	*b* (Å)	*γ*
6-6-6-6	0	18.50	16.03	90.00
5-7-5-7 to 5-6-5-7				
1	1.59	17.48	16.53	90.01
TS1[a]	6.81	17.35	16.39	91.81
I1	6.60	17.22	16.35	91.33
TS2[a]	8.51	17.35	16.39	91.81
2	4.40	17.22	16.25	93.61
TS3[a]	8.05	17.50	16.11	94.64
I3	5.82	17.53	16.01	95.01
TS4[a]	6.83	17.50	16.11	94.64
3	1.88	17.77	15.98	95.68
5-7-5-7 to 5-7-6-7				
1	1.59	17.48	16.53	90.01
TS1[a]	6.50	17.51	16.26	89.33
I1	6.09	17.50	16.02	90.04
TS2[a]	7.25	17.51	16.26	89.33
2	3.27	17.53	15.98	88.66
TS3[a]	6.99	17.59	16.03	86.94
I3	5.73	17.59	16.03	86.94
TS4[a]	6.26	17.59	16.03	86.94
3	1.44	17.64	16.07	85.21
5-7-5-7 to 5-8-5-8				
1	1.59	17.48	16.53	90.01
TS1[a]	11.50	17.16	16.66	90.89
TS2[a]	10.70	16.63	17.07	90.82
2	4.05	16.84	16.78	91.77
3	1.18	16.43	17.37	89.88

[a] Cell parameters for transition states are not optimized, the average of the cell parameters of the initial and final states is used.

Charge Density Analysis

The planar averaged charge density profile of the SiO_2/Ru(0001) system is given by integrating the surface area of the supercell along a given direction (z-axis is used)

$$\rho_z(z) = \iint \rho(x, y, z) dxdy, \tag{1}$$

with $\rho_z(z)$ having unit of $e/\text{Å}$.

Charge density difference was calculated as follows:

$$\Delta\rho_z(z) = \rho_{z_{SiO_2}}(z) + \rho_{z_{Ru}}(z) - \rho_{z_{SiO_2/Ru}}(z), \tag{2}$$

where $\rho_{z_{SiO_2}}(z)$, $\rho_{z_{Ru}}(z)$, and $\rho_{z_{SiO_2/Ru}}(z)$ are the charge density of the SiO_2 system, Ru system, and total SiO_2/Ru(0001) system, respectively. Accordingly, the negative sign of $\Delta\rho_z(z)$ indicates electron accumulation, whereas a positive sign denotes electron depletion.

The charge transfer between the SiO_2 bilayer and the Ru(0001) substrate was calculated as follows:

$$\Delta q_{SiO_2} = \int_{z_{mid}}^{L_z} \Delta\rho_z(z)\, dz, \tag{3}$$

where z_{mid} denotes the middle interface position ≈ 12.2Å between the SiO_2 bilayer and the Ru(0001) substrate (see Figure S3), which also corresponds to the minimum of the planar averaged charge density $\rho_z(z)$. L_z denotes the upper limit of integration along z-axis direction.

According to our definition, all calculated values of Δq_{SiO_2} are positive, which means that the electrons flow from the SiO_2 bilayer to the Ru(0001) substrate from overall view.

According to our definition, a positive value of charge transfer (Δq_{SiO_2}) indicates a net electron transfer from the SiO_2 bilayer to the Ru(0001) substrate. All calculated values of Δq_{SiO_2} are positive, confirming that, overall, electrons flow from the bilayer to the substrate.

Planar averaged charge density profiles were calculated using Cube Suite,[2] and charge density differences were visualized using VESTA.[3]

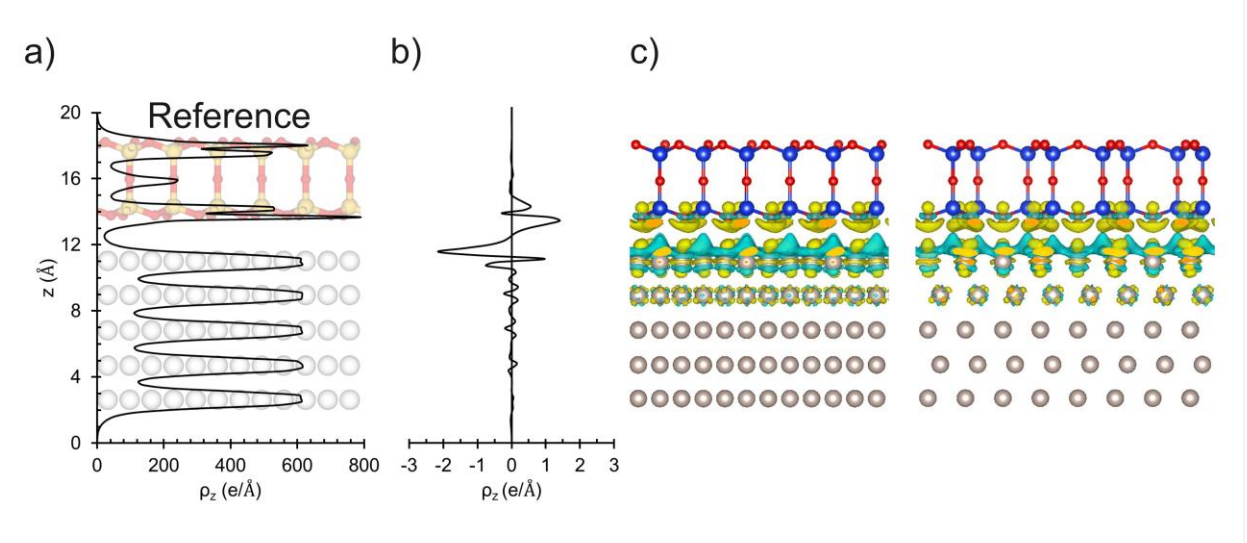

Figure S3. Charge density analysis of the SiO_2/Ru(0001) with a 6-6-6-6 ring system (reference structure). a) Planar averaged charge density profile of the SiO_2/Ru(0001) system along z-axis. b) Planar averaged charge density difference profile along z-axis. c) Charge density difference plots with side views along the x- and y-axis. (cyan: electron accumulation, yellow/green: electron depletion).

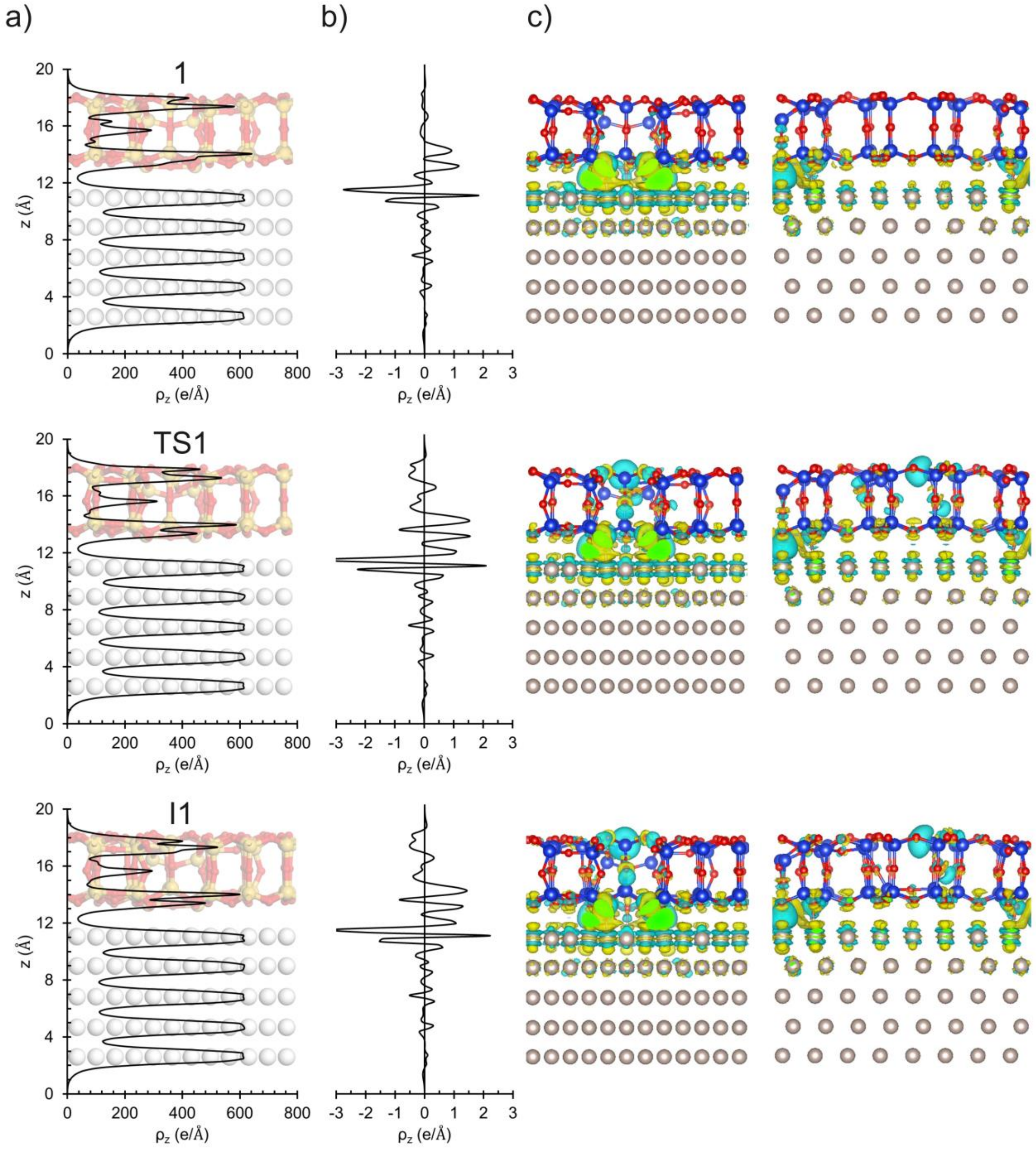

a)
b)
c)
1
TS1
I1
z (Å)
ρz (e/Å)

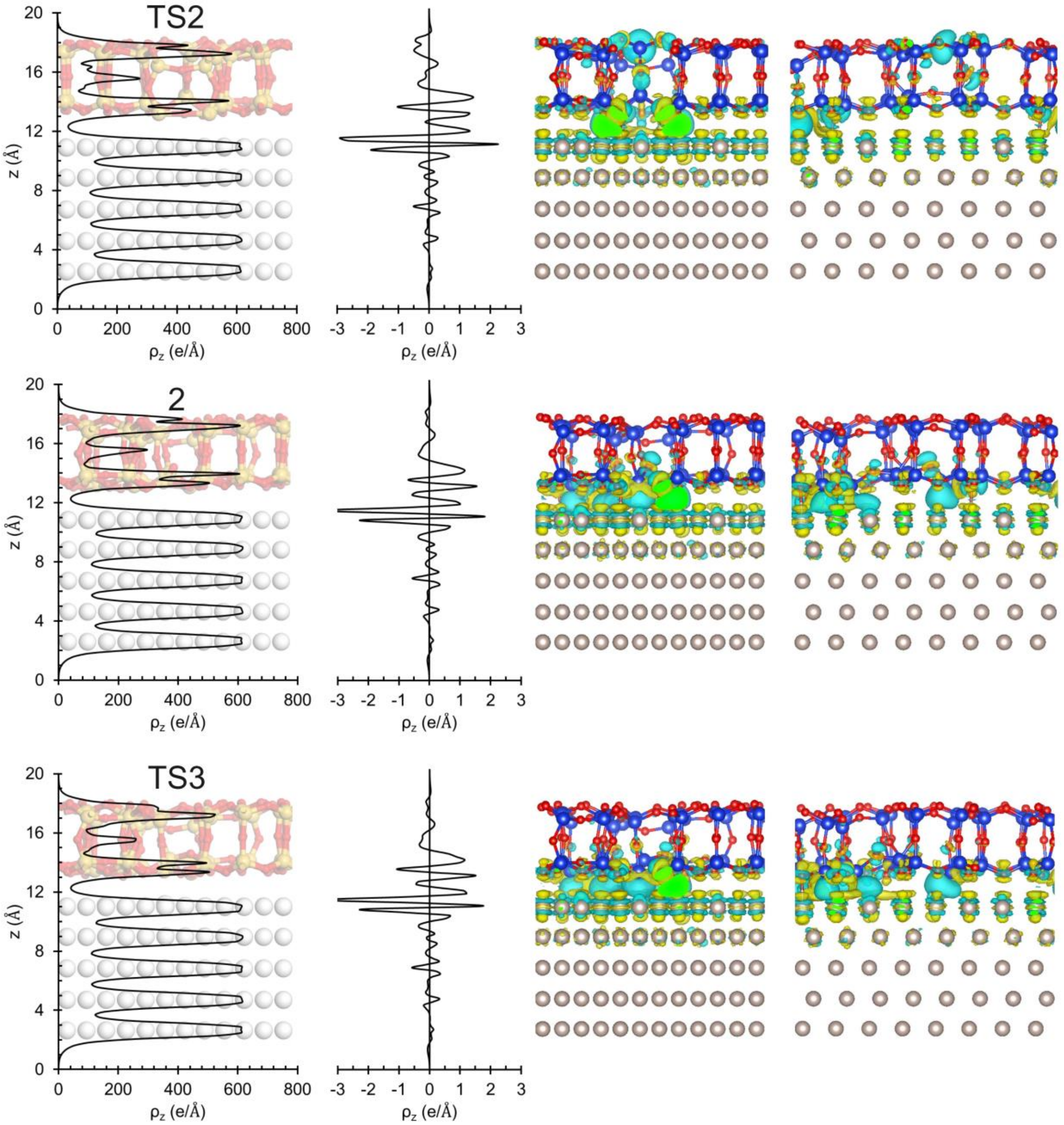
TS2
2
TS3
z (Å)
ρz (e/Å)
0
4
8
12
16
20
0
200
400
600
800
-3
-2
-1
0
1
2
3

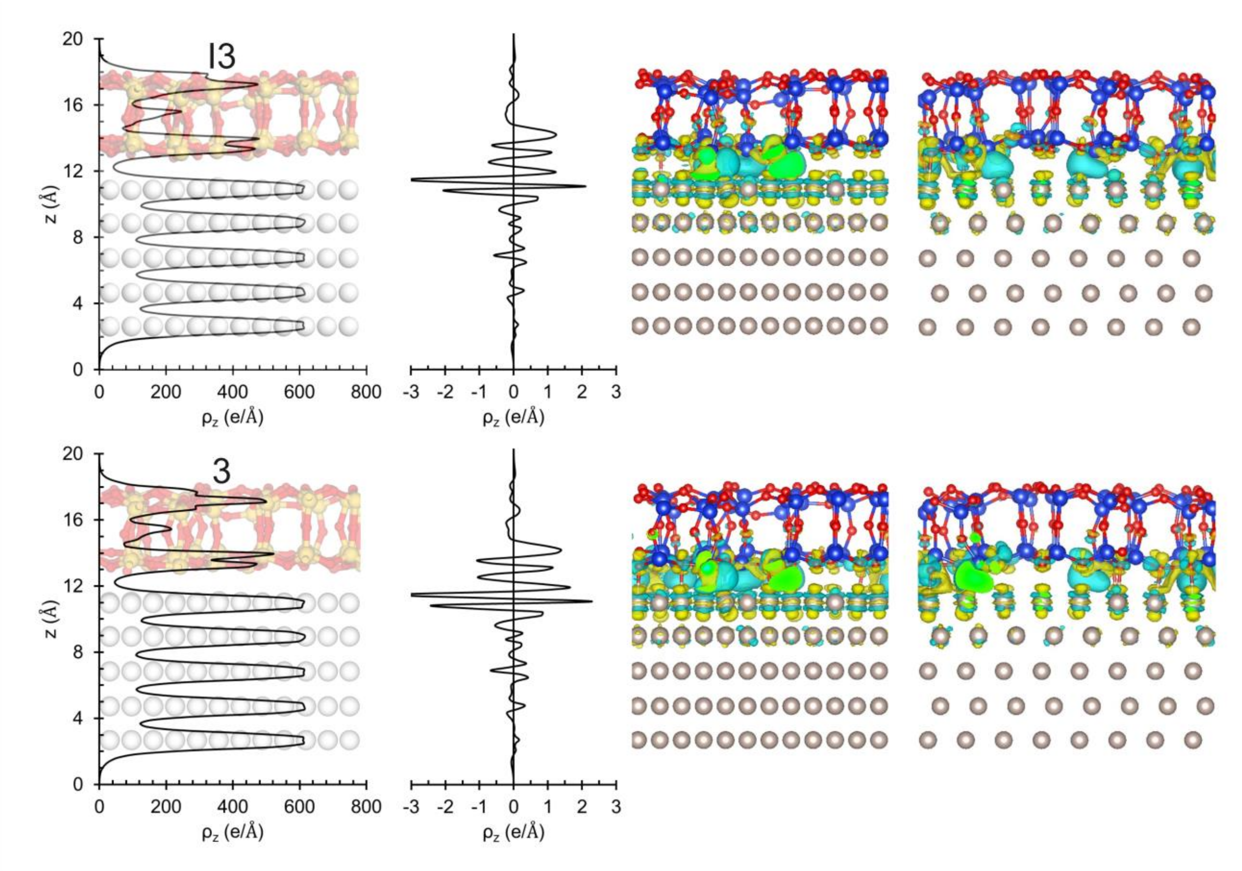

Figure S4. Charge density analysis of the SiO_2/Ru(0001) from a 5-7-5-7 to a 5-6-5-7 ring system. a) Planar averaged charge density profiles of the SiO_2/Ru(0001) system along z-axis. b) Planar averaged charge density difference profiles along z-axis. c) Charge density difference plots with side views along the x- and y-axis. (cyan: electron accumulation, yellow/green: electron depletion).

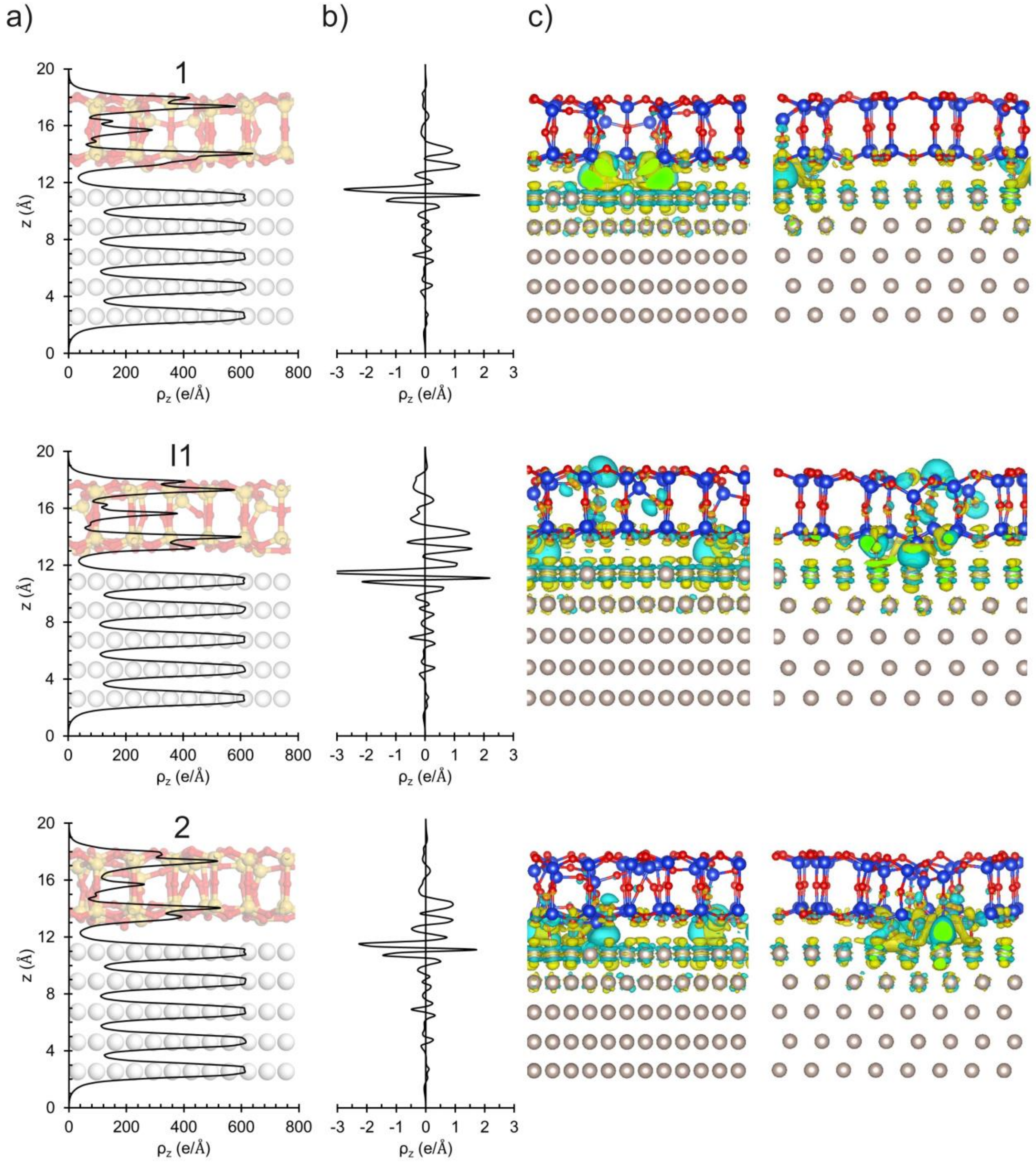

a)
b)
c)
1
I1
2
z (Å)
ρ_z (e/Å)
0
4
8
12
16
20
0 200 400 600 800
-3 -2 -1 0 1 2 3

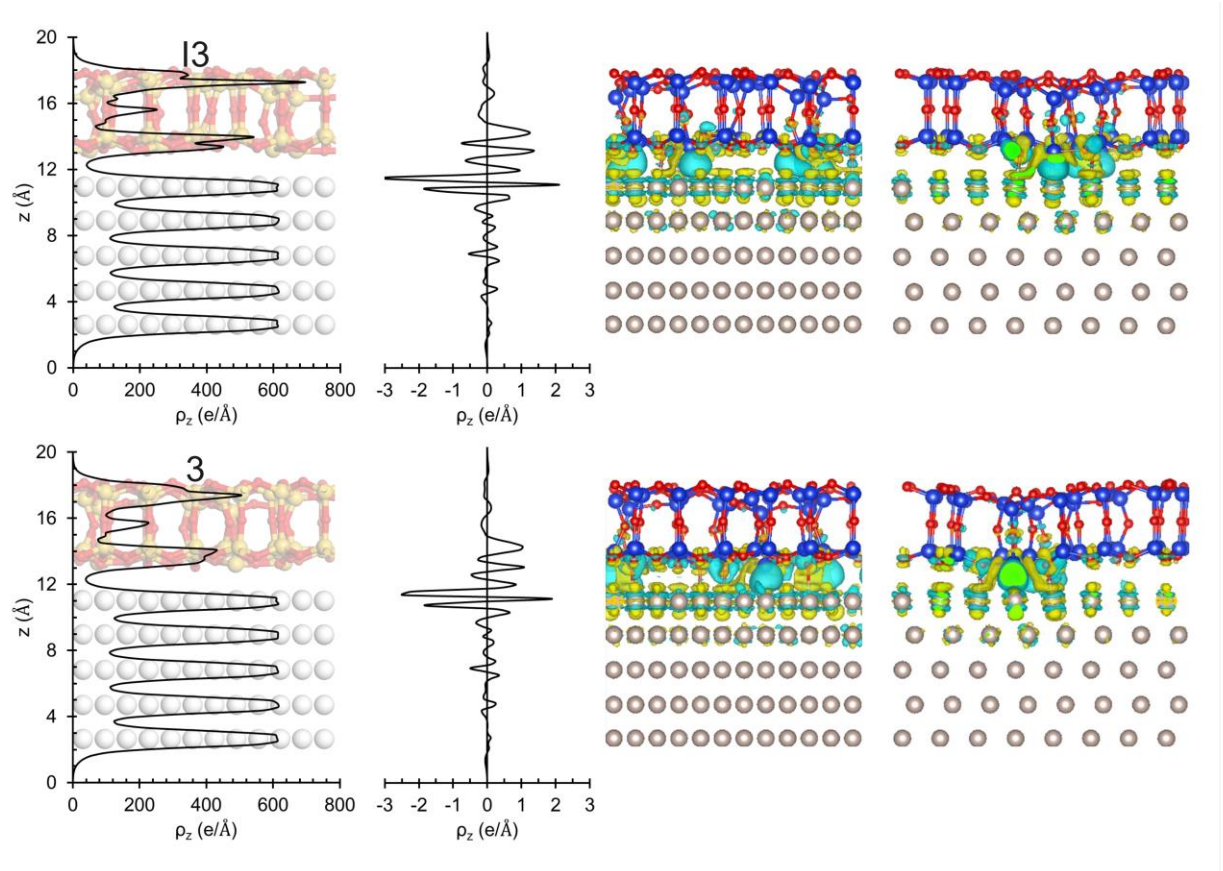

Figure S5. Charge density analysis of the SiO_2/Ru(0001) with stable and intermediate structures from a 5-7-5-7 to a 5-7-6-7 ring system. a) Planar averaged charge density profiles of the SiO_2/Ru(0001) system along z-axis. b) Planar averaged charge density difference profiles along z-axis. c) Charge density difference plots with side views along the x- and y-axis. (cyan: electron accumulation, yellow/green: electron depletion).

Planar Averaged Charge Density Profiles in Z Direction

To evaluate whether the STM tip–SiO_2 bilayer interaction could influence our imaging, we computed planar averaged charge density profiles along the surface normal for pure Ru(0001) and for the entire SiO_2/Ru(0001) system after subtracting the density of the SiO_2 bilayer. This difference profile isolates the metal contribution that spills through the oxide. The profiles are overlaid on a side view of the 6-6-6-6 ring system on the Ru(0001) substrate with a common z-axis scale (Figure S6). Because of the finite vacuum and grid, the analysis extends to ~0.25 nm above the top-O plane of the SiO_2 bilayer.

Under typical tunneling conditions, no structural changes have been observed in previous low-temperature STM measurements. The new STM experiments at room temperature were performed using similar tunneling parameters (V_S = 0.8 V, I_T = 1 nA). For such setpoints, the STM tip–surface separation is estimated to be ~0.5 nm[4–6]. It is also established that metal wave functions can spill out through thin oxide films[7], which is ensuring that the tunneling conditions are maintained

For the model shown in Figure S6, the geometric parameters are: d_{Si-Ru} = 3.24 Å, d_{Si-O} = 1.64 Å, and δ_{O-Si} = 0.57 Å. The distance from the Ru top layer to the top-O plane is approximately 0.71 nm.

When the two profiles are overlaid on a common z axis, they become essentially identical from about 0.15 nm above the SiO_2 surface and upward, and then decay smoothly. In plain terms, the electron density that the tip senses above the bilayer looks the same as for a pure Ru surface. The tunnelling signal is dominated by metallic states that spill through the bilayer, not by states localized in SiO_2. This means our imaging does not require pressing the tip untypical close to the SiO_2 bilayer, which makes strong tip–film interactions unlikely under our conditions.

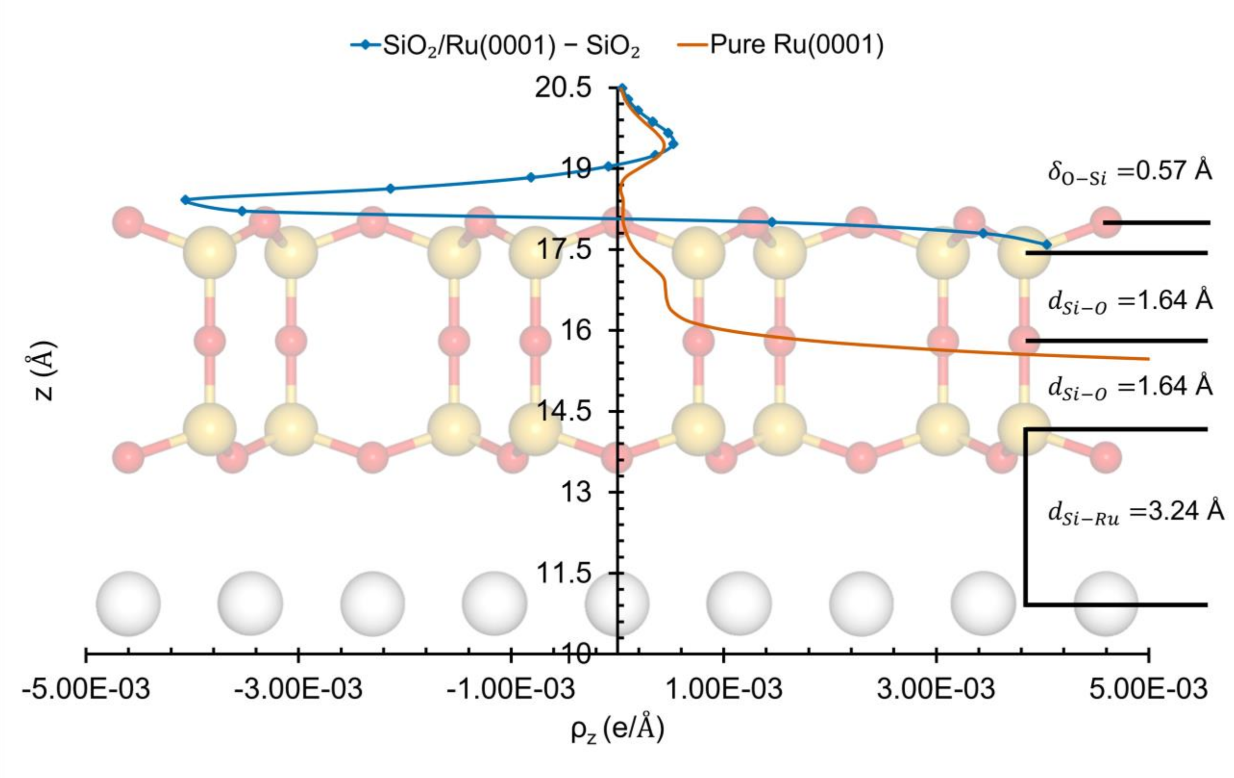

Figure S6. Planar averaged charge density profiles above a SiO_2/Ru(0001) with a 6-6-6-6 ring system. Blue: Difference profile (SiO_2/Ru – SiO_2), highlighting the Ru contribution through the oxide. Orange: Pure Ru(0001) reference. Above ~0.15 nm from the top-O plane the curves coincide, indicating metallic spill-out through the bilayer. Structural parameters for the illustrated model: d_{Si-Ru} = 3.24 Å, d_{Si-O} = 1.64 Å, δ_{O-Si} = 0.57 Å; Ru-to-top-O height ≈ 0.71 nm. For clarity, points inside the film are omitted in the blue curve.

Experimental Section

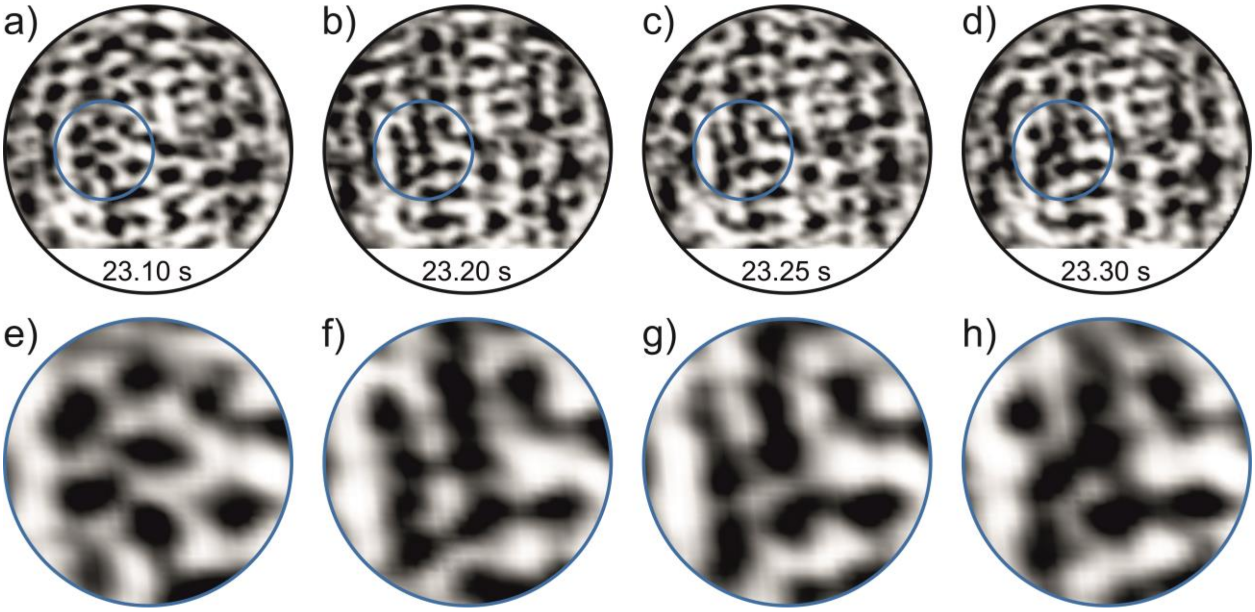

Figure S7. Dynamic changes of the apparent ring configuration. a-d) High-speed spiral STM images on 2D silica (V_S = 0.8 V, I_T = 1 nA, T = 300 K, scan diameter = 5 nm). Each frame is acquired within 50 ms. The absolute time is indicated at the bottom of the frames and is related to the supplementary video. The regions highlighted in blue are magnified in e-h).

The supplementary video shows the scan sequence from which the experimental figures in the main manuscript and Figure S7 are extracted. In addition, on the left, the motion magnified video runs simultaneously. The real time of the scan is provided at the bottom of the original video and corresponds to the times provided in the figures of the main manuscript and the supplement. The scan parameters are provided at the bottom of the video frame.

References

[1] H. W. Klemm, M. J. Prieto, F. Xiong, G. B. Hassine, M. Heyde, D. Menzel, M. Sierka, T. Schmidt, H.-J. Freund,"A Silica Bilayer Supported on Ru(0001): Following the Crystalline-to Vitreous Transformation in Real Time with Spectro-microscopy" *Angew. Chem. Int. Ed.* **2020**, 59, 10587-10593.

[2] Cube Suite - A web app (GUI) for Cube Toolz, can be found under https://cubesuite.streamlit.app/, **2024**.(accessed: 01. July 2025)

[3] K. Momma, F. Izumi,"VESTA: a three-dimensional visualization system for electronic and structural analysis" *J. Appl. Crystallogr.* **2008**, *41*, 653-658.

[4] C. J. Chen, "Introduction to Scanning Tunneling Microscopy" *Oxford University Press* **2021**.

[5] J. Tersoff, D. R. Hamann, *Physical review letters* **1983**, 50, 1998.

[6] J. M. Blanco, C. González, P. Jelínek, J. Ortega, F. Flores, R. Pérez, "First-principles simulations of STM images: From tunneling to the contact regime" *Phys. Rev. B* **2004**, 70, 085405

[7] G. Pacchioni, H. Freund, "Electron Transfer at Oxide Surfaces. The MgO Paradigm: from Defects to Ultrathin Films" *Chemical Reviews* **2013**, 113, 4035.
